# Using Elevated Cholesterol Synthesis as a Prognostic Marker in Wilms' Tumor: A Bioinformatic Analysis

**DOI:** 10.1155/2021/8826286

**Published:** 2021-01-28

**Authors:** Yuanbin He, Xu Cui, Yu Lin, Yunjin Wang, Dianming Wu, Yifan Fang

**Affiliations:** Department of Pediatric Surgery, Fujian Maternity and Child Health Hospital, Affiliated Hospital of Fujian Medical University, Fuzhou 350001, China

## Abstract

**Background:**

Wilms tumor is the most common renal malignancy of children. Identifying factors that could predict the prognosis of patients with Wilms tumor is clinically meaningful. Many studies found tumors with elevated cholesterol synthesis that are featured with dismal prognosis. Even in some clinical trials, people with excessive dietary cholesterol intake and high plasma low-density lipoprotein levels are observed to have increased risk for cancer. However, the role of cholesterol biosynthesis in Wilms tumor has not yet been well clarified.

**Methods:**

RNA sequencing transcriptome data and all corresponding clinicopathological information used in our study were downloaded from the TARGET database. High-throughput sequencing (Fragments Per Kilobase of transcript per Million fragments mapped) data sets of 130 tumor samples and 6 normal samples were obtained for further analysis.

**Results:**

Wilms tumor samples with higher activity of cholesterol synthesis are characterized with worse overall survival (*P* < 0.05). In addition, Wilms tumor samples with mitigated activity of cholesterol synthesis are featured with better dendritic cell (DC) function and cytolytic activity (*P* < 0.05). Furthermore, we constructed a prognosis model based on differential expressed cholesterol synthesis-related genes (DECSG), which could predict the OS of patients with Wilms tumor accurately. KEGG and GO analysis of differential expressed genes between tumor samples with high and low cholesterol synthesis indicated that DECSGs are highly enriched in “mitosis nuclear division,” “nuclear division,” “chromosome segregation,” “cell cycle,” “Spliceosome,” and “RNA transport.”

**Conclusions:**

In conclusion, our study reported increased cholesterol synthesis in Wilms tumor predicts a worse prognosis and mitigated cytolytic activity, DC function, and MHC I signature in the tumor microenvironment. We also constructed a prognosis model for predicting the OS of patients with good accuracy, which is promising in clinical translation. Future studies should focus on the detailed mechanism that caused increasing cholesterol which promotes tumor progression and undermines patients' survival.

## 1. Introduction

Wilms tumor is the most common renal malignancy of children [1]. Although most of the patients have promising prognosis thanks to the advances in modern treatment modalities, 10% of the children with Wilms tumor still die of this disease [1–3]. Hence, the identification of factors that could predict the prognosis of patients with Wilms tumor is essential in daily clinical practice.

The accumulation of cholesterol is a general feature of cancer tissues [4, 5]. Many studies found tumors with elevated cholesterol synthesis that are featured with poor prognosis [6, 7]. Even in some clinical trials, people with excessive dietary cholesterol intake and high plasma low-density lipoprotein levels are observed to increase the risk for cancer occurrence [8–11]. However, the role of cholesterol biosynthesis in Wilms tumor has not yet been well clarified.

Transcriptome sequencing has become a silver bullet to identify genetic alterations in various malignancies [12–14], which dramatically enriched our knowledge of tumor biology. For example, Kandimalla et al. developed an accurate prognosis model based on the transcriptome expression of 15 immune, stromal, and proliferation gene signatures, facilitating clinicians to predict the survival time of their patients and adjust treatment modalities [15]. Similarly, a signature consisted of glycolysis-related genes was screened to predict the metastasis and survival of patients with lung adenocarcinoma [16]. However, a prognostic model based on transcriptome sequencing of Wilms tumor has rarely been reported.

In this context, we intend to investigate whether elevated cholesterol synthesis in the transcriptome level might have potential impact on the worse prognosis of Wilms tumor. In addition, a recent pilot study revealed an immune-engaged tumor microenvironment that exists within Wilms tumor, suggesting that Wilms tumor may be susceptible to immunotherapy as adult renal malignancies do [17]. Therefore, we also explored the relationship between cholesterol synthesis in Wilms tumor and immune microenvironment in the present study, which could provide more valuable information for future clinical studies.

## 2. Methods

### 2.1. Dataset Acquisition

RNA sequencing transcriptome data and all corresponding clinicopathological information used in our study were downloaded from the TARGET database (https://ocg.cancer.gov/programs/target), and the gene expression matrix were presented in the supplementary material [18]. High-throughput sequencing (Fragments Per Kilobase of transcript per Million fragments mapped, FPKM) data sets of 130 tumor samples and 6 normal samples were obtained for further analysis: FPKM = FPKM = total exon fragments/(mapped reads (Millions) × exon length (KB)).

### 2.2. Statistical Analysis

### 2.3. Evaluate the Activity of Cholesterol Synthesis in Wilms Tumor

First, we acquired the genes involved with cholesterol synthesis from The Molecular Signatures Database (MSigDB) (https://www.gsea-msigdb.org/gsea/msigdb/index.jsp), which is a collection of annotated gene sets for use with gene set enrichment analysis (GSEA) software [19]. The genes involved with cholesterol synthesis were listed as follows: ACAT2, ARV1, CYP51A1, DHCR24, DHCR7, EBP, FDFT1, FDPS, GGPS1, HMGCR, HMGCS1, HSD17B7, IDI1, IDI2, LBR, LSS, MSMO1, MVD, MVK, NSDHL, PLPP6, PMVK, SC5D, SQLE, and TM7SF2 (Table 1). Then, we calculated the relative activity of cholesterol synthesis, referred to as Chole_score by single sample gene set enrichment analysis (ssGSEA) of each tumor sample [20]. Furthermore, we divided samples into Chole_high and Chole_low groups based on the quartile of cholesterol synthesis score. ssGSEA was conducted using *R* package “GSVA” and “GSEAbase” [21].

### 2.4. Perform Survival Analysis and Cumulative Hazard Analysis for Wilms Tumor with Different Activity of Cholesterol Synthesis

Kaplan-Meier (KM) analysis was performed to determine survival outcomes. The top and bottom 20% patients were compared in terms of the cholesterol scores using the KM curve, and the statistical significance was evaluated by the log-rank test. Cumulative hazard at a specific time point (*H*(*t*)) is defined as “-log(*S*(*t*))”, where *S*(*t*) means the survival probability at a specific time point. The *R* packages to perform survival analysis and cumulative hazard analysis are “survival” and “survminer” [21].

### 2.5. Explore Differentially Expressed Genes between Tumor Samples with High and Low Cholesterol Synthesis Activity

First, the *R* package of “Limma” was used to screen differentially expressed genes [22]. Second, we used Cox proportional hazard regression analysis to evaluate the association of the differentially expressed genes in predicting overall survival (OS) in patients with Wilms tumor. Third, we selected OS-related genes and performed lasso regression on these genes. Then, Wilms tumor samples are divided into two groups with high and low lasso risk based on the expression level of these OS-related genes. The KM curve was plotted to visualize the survival difference between two lasso risk-based groups. The ROC curve was depicted to assess the accuracy of the model we constructed using lasso regression. The *R* package for Cox proportional hazard regression, lasso regression, and survival analysis is “survival.” The *R* package for the ROC curve is “survivalROC” [23]. We performed Kyoto Encyclopedia of Genes and Genomes (KEGG) and Gene Ontology (GO) analysis to annotate the potential function of the differential expressed genes. The *R* package “http://org.Hs.eg.db” was used to provide annotation information of genes [24].

### 2.6. Investigate the Relationship between the Activity of Cholesterol Synthesis of Wilms Tumor and 29 Immune Signatures

First, we estimated the infiltration of immune cells of each sample using T to Estimate the Proportion of Immune and Cancer cells (EPIC). Second, we performed ssGSEA for 130 Wilms tumor samples based on 29 immune signatures. Then, we compared the difference of immune signatures between tumor samples with high and low activity of cholesterol synthesis using Student's *t*-test. All statistical data and figures were analyzed using *R* software (version 3.6.2), SPSS 23 (IBM, Chicago, USA), and GraphPad Prism 7.04 (GraphPad Software, San Diego, USA). Results with *P* < 0.05 were considered statistically significant. All the raw code used in this study was listed as supplementary methods.

## 3. Results

### 3.1. Wilms Tumor Samples with Higher Activity of Cholesterol Synthesis Are Characterized with Worse OS Period

**Figure 1:**
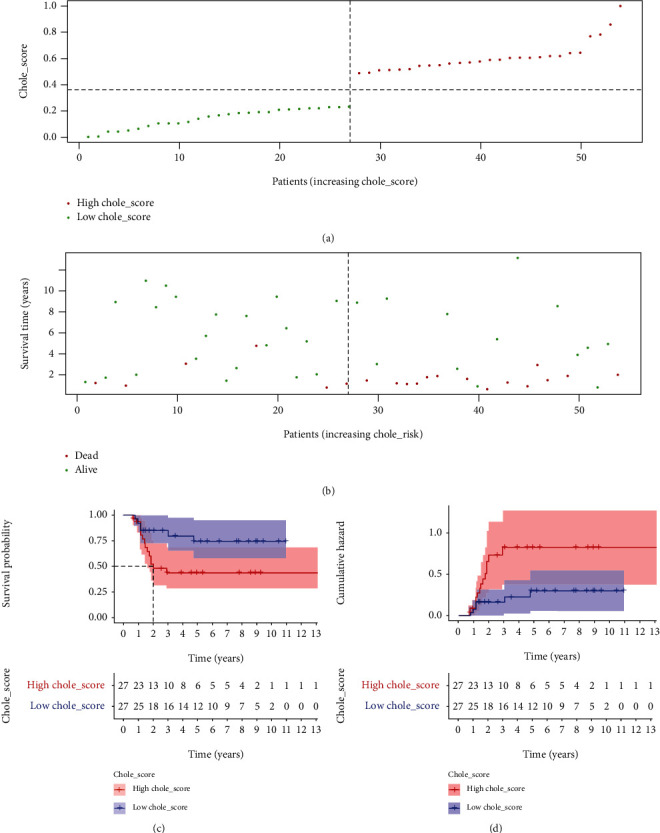
Wilms tumor samples with higher activity of cholesterol synthesis are characterized with worse overall survival. (a) Patients with Wilms tumor were divided into two groups (Chole_high and Chole_low) based on the cholesterol synthesis score. (b) The distribution of the survival status of patients with Wilms tumor along with increased Chole_score. (c) Survival analysis showed that Wilms tumors with decreased Chole_score were featured with prolonged survival time. (d) Cumulative hazard analysis showed that Wilms tumors with increased Chole_score were featured with expanded risk for worse survival.

Patients with Wilms tumor were divided into two groups (Chole_high and Chole_low) based on the cholesterol synthesis score, which was calculated by using ssGSEA (Figure 1(a)). The baseline characteristics of the patients were summarized in the supplementary Table 1. The raw data of gender, subtypes, and stage for 130 patients with Wilms tumor was summarized as supplementary Table 2. At a specific time point, more patients with higher cholesterol synthesis in their tumor tissue were dead (Figure 1(b)), suggesting the unfavorable role of excessive cholesterol synthesis to patients prognosis. To further confirm this hypothesis, we conducted a survival analysis and plotted a KM curve. The results showed that patients with higher cholesterol synthesis are featured with worse overall survival (*P* < 0.05). It is worth mentioning that the median survival time of these patients was only two years (Figure 1(c)), which was consistent with the cumulative hazard analysis (Figure 1(d)).

### 3.2. Wilms Tumor Samples with Mitigated Activity of Cholesterol Synthesis Are Featured with Better Dendritic Cell (DC) Function and Cytolytic Activity

**Figure 2:**
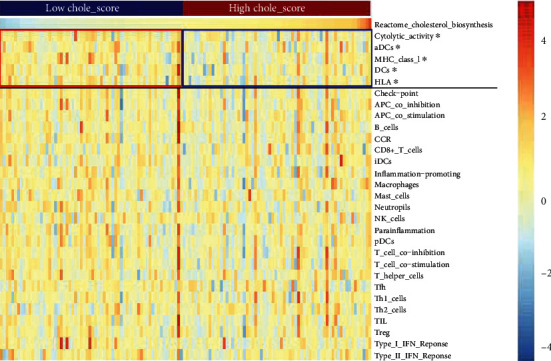
Wilms tumor samples with mitigated activity of cholesterol synthesis are featured with better dendritic cell (DC) function and cytolytic activity.

Immunotherapy is the hope of modern cancer therapy; however, the feasibility of immunotherapy in treatment of Wilms tumor was rarely discussed. Hence, we first estimated the infiltration level of immune cells in each sample of Wilms tumor. The results showed that the majority of Wilms' tumor tissue harbored 20% nontumor cells in its microenvironment, suggesting that Wilms tumor may be susceptible to immunotherapy as adult renal malignancies do (Supplementary Figure 1). Furthermore, we investigated whether the activity of cholesterol synthesis could affect immune signatures in a bulk tumor tissue. We found that the decreased score of cholesterol synthesis was paralleled with higher cytolytic activity, DCs function, MHC I, and HLA (Figure 2). We also found that the score of cholesterol synthesis can divide aDCs, cytolytic activity, DC, MHC I, and HLA all into two groups (Supplementary Figure 2; *P* < 0.05). Given that tumor samples with the mitigated activity of cholesterol synthesis are characterized with better prognosis, enhanced anticancer immunity in the tumor microenvironment of Chole_high samples might be a reasonable explanation.

### 3.3. A Prognosis Model Based on Differential Expressed Cholesterol-Synthesis-Related Genes (DECSG) Could Predict the OS of Patients with Wilms Tumor

**Figure 3:**
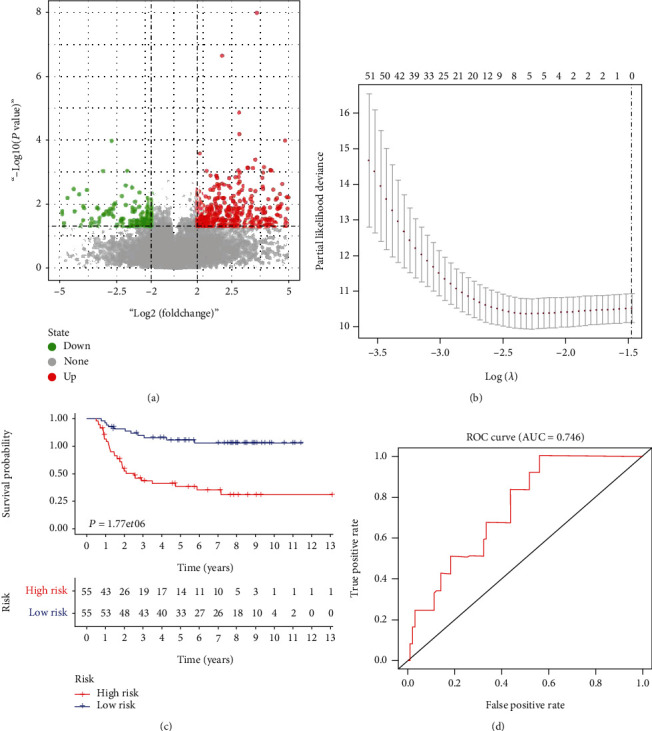
A prognosis model based on differential expressed cholesterol synthesis-related genes (DECSG) could predict the OS of patients with Wilms tumor. (a) Volcano plot presented differentially expressed genes between Chole_high and Chole_low groups. (b) Lasso regression kept 9 genes for the construction of the prognosis model based on the smallest partial likelihood deviance. (c) Survival analysis showed patients with low lasso risk have prolonged survival time. (d) The ROC curve indicated a good accuracy of the prognosis model based on cholesterol synthesis.

First, we identified 411 differential expressed genes between Chole_high and Chole_low groups (Figure 3(a)). Then, we evaluated the association between each DECSG expression and the OS of patients with Wilms tumor. A total of 63 (15.3%) genes were associated with patients' OS. We further conducted a lasso regression to eliminate some genes, which may lead to overfitting phenomenon. Finally, we constructed a prognosis model with nine left genes according to partial likelihood deviance (Figure 3(b)). Each patient was labeled with a lasso risk given by the constructed prognosis model. Survival analysis showed patients with lower lasso risk were featured with prolonged OS ((Figure 3(c); *P* < 0.05). We further depicted the ROC curve to assess the accuracy of the constructed prognosis model. The results demonstrated this model harbored a good predictive accuracy (area under curve (AUC) = 0.746), suggesting it has a promising value of clinical translation (Figure 3(d)).

### 3.4. KEGG and GO Analysis of Differential Expressed Genes between Tumor Samples with High and Low Cholesterol Synthesis Score

**Figure 4:**
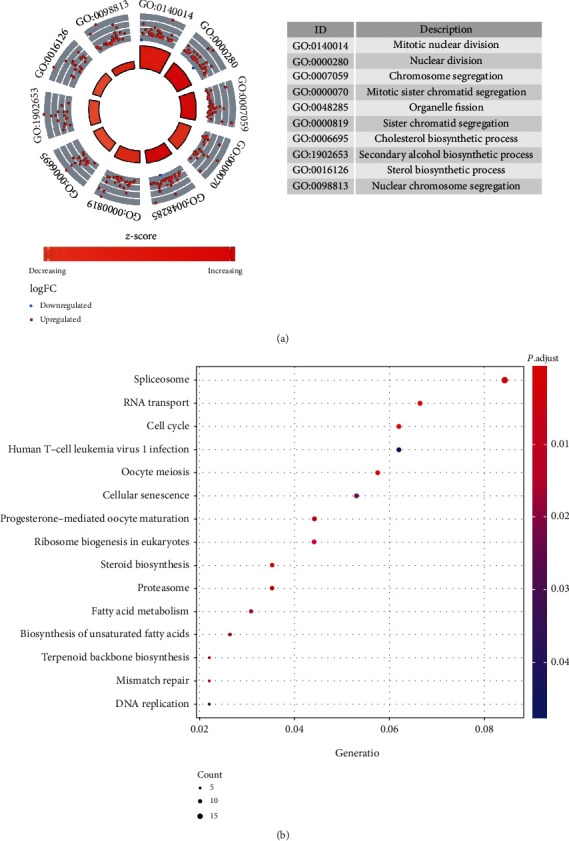
KEGG and GO analysis of differential expressed cholesterol synthesis-related genes.

Although the constructed prognosis model showed promising clinical implications, the detailed function of these DECSG is still not clear. Hence, we conducted KEGG and GO analysis to annotate their related signal pathways and potential functions. The GO results showed that more cell division-associated genes were upregulated in tumor samples with increased cholesterol synthesis, such as “mitosis nuclear division,” “nuclear division,” and “chromosome segregation”, suggesting that activated cholesterol synthesis may contribute to the proliferation of Wilms tumor cells (Figure 4(a)). Similarly, KEGG analysis demonstrated that DECSGs are highly enriched in “cell cycle,” “Spliceosome,” and “RNA transport”, which are commonly activated in tumor development. An interesting finding was genes involved with “DNA repair” were upregulated in tumor samples with increased cholesterol synthesis, which implicated that tumor cells may utilize enhanced cholesterol synthesis to improve DNA repair system and further lead to chemotherapy resistance (Figure 4(b)).

## 4. Discussion

Although the majority of patients with Wilms tumor have promising prognosis benefited from medical advances, 10% of all patients still die of this disease. Hence, early identification of patients with dismal prognosis will improve treatment stratification, which might lead to reduction of the direct and late effects of chemotherapy. The present study demonstrated that excessive cholesterol synthesis in the Wilms tumor tissues is associated with worse OS of patients. Besides, more samples with low activity of cholesterol synthesis were FHWT (favorable histology), while samples with high activity of cholesterol synthesis were DAWT(cellular diffuse anaplasia), suggesting there might be an association between cholesterol synthesis and different subtypes of WT. In addition, a prognosis model based on the expression level of DECSGs was developed with good accuracy (AUC = 0.746).

Cholesterol synthesis starts with the conversion of citrate, derived from the tricarboxylic acid (TCA) cycle in the mitochondria, to acetyl coenzyme A (acetyl-CoA), followed by a cascade of enzymatic reactions in the endoplasmic reticulum known as the mevalonate pathway, where acetyl-CoA is converted to lanosterol [25]. Excessive cholesterol synthesis fueled tumor progression through multiple approaches. On one hand, cholesterol is an essential precursor of estrogen and androgen, high level of which are associated with an increased risk of breast and prostate cancer, respectively [26, 27]. On the other hand, cholesterol is a major component of lipid rafts that regulate cancer cell migration and invasion. Numerous reports have demonstrated that CD44 is located in lipid rafts and contributed to tumor progression [28, 29]. Decreased cholesterol results in disordered CD44 localization, raft-dependent CD44 shedding, and the suppression of tumor cell migration [30]. Decreased cholesterol also enhances the CD44-mediated adhesion of lymphocytes, suggesting that lipid rafts regulate lymphocyte interactions under physiological flow conditions [30]. In the present study, we performed KEGG and GO analysis to explore the related signal pathways and potential functions of DECSGs. The results revealed a potential association between increased cholesterol synthesis and cell division, especially nuclear division, but the underlying mechanism of the altered cholesterol metabolism which affects the cell division is yet to be discovered.

Many studies correlated the tumor cell metabolism with the altered immune microenvironment [31–34]. For example, mounting evidence suggested that tumor glycolysis also played a significant role in forming immunosuppressive networks that are important for cancer cells to escape immune surveillance (“immune evasion”) [35]. Extracellular accumulation of lactate derived from glycolysis alters the TME by generating acidic pH, which is detrimental to immune cells. Such low-pH TME has been reported to suppress the physiology of cytotoxic and antigen-presenting cells [36]. Additionally, cancer cells have been known to modulate the metabolic phenotype of cancer-associated factors from oxidative phosphorylation to glycolysis and vice versa [37–39]. Similarly, many studies have investigated the influence of cholesterol on the anticancer immunity. The metabolite of cholesterol, 27-hydroxycholesterol, functions as a biochemical mediator of the metastatic effects of hypercholesterolemia in breast cancers through its actions on *γδ*-T cells and polymorphonuclear neutrophils. ATP-binding cassette transporter G1 (ABCG1) promotes cholesterol efflux from cells and regulates intracellular cholesterol homeostasis [40]. Sag et al. reported that deletion of *ABCG1* dramatically suppressed subcutaneous bladder carcinoma and melanoma growth and prolonged survival [40]. They also showed that reduced tumor growth by deleting *ABCG1* is myeloid cell intrinsic and is associated with a phenotypic shift of the macrophages from a tumor-promoting M2 to a tumor-fighting M1 within the tumor [40]. In our study, samples with low cholesterol synthesis were featured with higher levels of cytolytic activity, DC function, and MHC I signature, suggesting that low cholesterol synthesis was associated with robust anticancer immunity in Wilms tumor. Very recently, a well-conducted study reported inhibition of PCSK9, a key enzyme upregulates the cholesterol synthesis, that could enhance the antitumor immunity in the tumor microenvironment, which partially confirmed our results.

The present study has some strengths to declare. First, it is the first study to report a role of cholesterol synthesis in Wilms' tumor prognosis, which filled a blank in the landscape of the correlated metabolism with tumor development. Second, we developed a prognosis model that is of great accuracy to predict patients' OS, which could be utilized in clinical practice. Third, we correlated lower activity of cholesterol synthesis with the robust anticancer immunity for the first time in Wilms tumor. Given these findings, we could image the feasibility of using inhibitors of cholesterol synthesis like statins to treat Wilms tumors. In fact, many clinical trials have investigated whether the use of statins could affect cancer prevalence, progression, and relapse [41–44]; however, the results were still inconclusive. Another obstacle limited to the efficacy of statins in cancer therapy was the local dosage in tumor tissues that may not be sufficient to interfere the cholesterol synthesis of tumor cells. Hence, acknowledging how much dosage of statins can inhibit the growth of cancer cells is a topic worth studying in future experiments. Besides, a combination of statins and immune checkpoint inhibitors could also be launched in future clinical trials because we revealed a negative relationship between cholesterol synthesis and robust anticancer immunity. Nevertheless, this study also has many limitations to confess. First, this is an in silico study that lacks of validations from laboratory experiments and other published cohorts due to no other RNA sequence data that could be acquired except for the TARGET cohort. Second, a common shortcoming of bioinformatic studies is a lack of mechanism exploration, especially the approach of how increased cholesterol synthesis in tumor cells determined worse prognosis for patients with Wilms tumor. Third, the clinical information in the TARGET database lacks many valuable factors for WT prognosis like postoperative complications; however, these factors may not be confounding of the present study but the resultant phenomenon caused by the variation of intratumoral cholesterol synthesis. For example, more DAWT cases were presented in chole_high samples, and it is not clear whether overactivation of cholesterol synthesis mediated DAWT and worse survival, or the observed survival benefits in the chole_low group were just caused by the inequality of distribution of pathological tissue types.

In conclusion, our study found that elevated cholesterol synthesis indicated a worse prognosis and mitigated cytolytic activity, DC function, and MHC I signature in the tumor microenvironment in Wilms tumor. Furthermore, we constructed a prognosis model with good accuracy to predict the OS of patients, which is useful in the clinical translational prospect. Future studies should focus on the underlying mechanism of the increased cholesterol synthesis, which helps to reveal the mask that causes tumor progression and shortens patients' survival.

## Figures and Tables

**Table 1:** The full name of cholesterol_synthesis genes.

Gene_symbol	Full_name
ACAT2	Hydroxysteroid 17-beta dehydrogenase 7
ARV1	ARV1 homolog, fatty acid homeostasis modulator
CYP51A1	Cytochrome P450 family 51 subfamily A member 1
DHCR24	24-Dehydrocholesterol reductase
DHCR7	7-Dehydrocholesterol reductase
EBP	Cholestenol delta-isomerase
FDFT1	Farnesyl-diphosphate farnesyltransferase 1
FDPS	Farnesyl diphosphate synthase
GGPS1	Geranylgeranyl diphosphate synthase 1
HMGCR	3-Hydroxy-3-methylglutaryl-CoA reductase
HMGCS1	3-Hydroxy-3-methylglutaryl-CoA synthase 1
HSD17B7	Hydroxysteroid 17-beta dehydrogenase 7
IDI1	Isopentenyl-diphosphate delta isomerase 1
IDI2	Isopentenyl-diphosphate delta isomerase 2
LBR	Lamin B receptor
LSS	Lanosterol synthase
MSMO1	Methylsterol monooxygenase 1
MVD	Mevalonate diphosphate decarboxylase
MVK	Mevalonate kinase
NSDHL	NAD(P) dependent steroid dehydrogenase-like
PLPP6	Phospholipid phosphatase 6
PMVK	Phosphomevalonate kinase
SC5D	Sterol-C5-desaturase
SQLE	Squalene epoxidase
TM7SF2	Transmembrane 7 superfamily member 2

## Data Availability

The data used to support the findings of this study are included within the article.

## Supplementary Materials

Supplementary Materials 1 The gene expression matrix was presented in the supplementary material. 2 All the raw code used in this study were listed as supplementary methods. 3 The baseline characteristics of the patients were summarized in the supplementary. Table 1 The raw data of gender, subtypes, and stage for 130 patients with Wilms tumor was summarized as supplementary Table 2.
