# Supplementary material for: Using Elevated Cholesterol Synthesis as a Prognostic Marker in Wilms' Tumor: A Bioinformatic Analysis
Source: Biomed Res Int. 2021 Jan 28;2021:8826286. doi: 10.1155/2021/8826286 (PMC7886595; doi:10.1155/2021/8826286)
Supplement: Supplementary Materials — 1 The gene expression matrix was presented in the supplementary material. 2 All the raw code used in this study were listed as supplementary methods. 3 The baseline characteristics of the patients were summarized in the supplementary. Table 1 The raw data of gender, subtypes, and stage for 130 patients with Wilms tumor was summarized as supplementary Table 2. [file 8826286.f1.zip › sTable1.docx]

|  |  | Chole_low | Chole_high | |
| --- | --- | --- | --- | --- |
| Stage | I | 12 (18.5%) | 5 (7.7%) | P = 0.12 |
|  | II | 28 (43.1%) | 25 (38.5%) |  |
|  | III | 21 (32.3%) | 25 (38.5%) |  |
|  | IV | 4 (6.2%) | 10 (15.4%) | P = 0.16 |
|  |  |  |  |  |
| Subtype | FHWT | 51 (78.5%) | 37 (56.9%) |  |
|  | DAWT | 14 (21.5%) | 28 (43.1%) | P = 0.014 |
|  |  |  |  |  |
| Sex | Male | 27 (41.5%) | 29 (44.6%) |  |
|  | Female | 38 (58.5%) | 36 (55.4%) | P = 0.86 |

Supplementary Table 1. The baseline clinical data of patients with high- and low Chole_scores.
