# Supplementary material for: Using Elevated Cholesterol Synthesis as a Prognostic Marker in Wilms' Tumor: A Bioinformatic Analysis
Source: Biomed Res Int. 2021 Jan 28;2021:8826286. doi: 10.1155/2021/8826286 (PMC7886595; doi:10.1155/2021/8826286)
Supplement: Supplementary Materials — 1 The gene expression matrix was presented in the supplementary material. 2 All the raw code used in this study were listed as supplementary methods. 3 The baseline characteristics of the patients were summarized in the supplementary. Table 1 The raw data of gender, subtypes, and stage for 130 patients with Wilms tumor was summarized as supplementary Table 2. [file 8826286.f1.zip › sTable2.docx]

sTable2. The gender, stage and subtypes of 130 wilms tumor

| ID | gender | stage | subtype |
| --- | --- | --- | --- |
| TARGET-50-PAKMUB | Male | II | FHWT |
| TARGET-50-PAJNLT | Female | IV | FHWT |
| TARGET-50-PAKYFC | Female | II | FHWT |
| TARGET-50-CAAAAM | Female | III | DAWT |
| TARGET-50-PAJNYT | Male | III | FHWT |
| TARGET-50-PAJNCJ | Female | II | FHWT |
| TARGET-50-PAJMVC | Male | III | DAWT |
| TARGET-50-PAJMEL | Female | IV | FHWT |
| TARGET-50-PAJNJJ | Female | II | FHWT |
| TARGET-50-PAJMKJ | Female | II | FHWT |
| TARGET-50-PAKECR | Female | II | DAWT |
| TARGET-50-PAEBXA | Female | III | FHWT |
| TARGET-50-PALGVY | Female | III | FHWT |
| TARGET-50-PAKZFK | Female | II | FHWT |
| TARGET-50-PAKZER | Female | II | FHWT |
| TARGET-50-PALJIP | Female | II | FHWT |
| TARGET-50-PAKZHF | Male | II | FHWT |
| TARGET-50-PAKRZW | Female | II | FHWT |
| TARGET-50-PAJLSP | Male | II | FHWT |
| TARGET-50-PAJMEN | Female | II | FHWT |
| TARGET-50-PAJMKN | Female | I | FHWT |
| TARGET-50-PAJNGH | Male | I | FHWT |
| TARGET-50-PAJNZU | Female | I | FHWT |
| TARGET-50-PAJPAU | Male | I | FHWT |
| TARGET-50-CAAAAS | Female | III | FHWT |
| TARGET-50-PAJPHA | Male | II | FHWT |
| TARGET-50-PAJMFY | Female | II | FHWT |
| TARGET-50-PAECJB | Female | III | FHWT |
| TARGET-50-PAJLTI | Male | I | FHWT |
| TARGET-50-PALGAZ | Female | I | FHWT |
| TARGET-50-PAJMSE | Female | III | FHWT |
| TARGET-50-PAKVET | Female | I | FHWT |
| TARGET-50-PAJNAV | Male | IV | FHWT |
| TARGET-50-PAJMIZ | Female | IIIB | FHWT |
| TARGET-50-PAJMMY | Male | III | FHWT |
| TARGET-50-PAJNTJ | Male | II | FHWT |
| TARGET-50-PAKSDG | Male | I | FHWT |
| TARGET-50-PAJNUP | Female | III | DAWT |
| TARGET-50-PAJLNJ | Female | II | FHWT |
| TARGET-50-PAJLKC | Female | II | DAWT |
| TARGET-50-PAJPDN | Female | II | DAWT |
| TARGET-50-PALKRS | Male | III | FHWT |
| TARGET-50-PALGLU | Female | III | FHWT |
| TARGET-50-PAJNNR | Male | III | DAWT |
| TARGET-50-PAJMUF | Male | III | FHWT |
| TARGET-50-PAJMLZ | Male | II | DAWT |
| TARGET-50-PAJMEP | Male | I | FHWT |
| TARGET-50-PAJNCZ | Male | I | FHWT |
| TARGET-50-PAKGED | Female | III | DAWT |
| TARGET-50-CAAAAR | Female | IV | FHWT |
| TARGET-50-PAJNDU | Male | III | FHWT |
| TARGET-50-PAKRCC | Male | I | DAWT |
| TARGET-50-PAEAFB | Male | III | FHWT |
| TARGET-50-PAJMXF | Male | I | FHWT |
| TARGET-50-PAJNNC | Male | II | FHWT |
| TARGET-50-PALLFB | Male | III | DAWT |
| TARGET-50-PAKULH | Female | IIIB | FHWT |
| TARGET-50-PALFME | Male | II | FHWT |
| TARGET-50-PAJLLF | Female | II | DAWT |
| TARGET-50-PAJNSL | Female | II | FHWT |
| TARGET-50-PAJNAA | Female | II | DAWT |
| TARGET-50-CAAAAO | Female | III | FHWT |
| TARGET-50-PAJPDC | Female | II | FHWT |
| TARGET-50-CAAAAP | Male | II | DAWT |
| TARGET-50-PAJNZK | Female | II | FHWT |
| TARGET-50-PALERC | Female | II | FHWT |
| TARGET-50-PAJLTH | Male | II | FHWT |
| TARGET-50-PAKSCC | Male | II | FHWT |
| TARGET-50-PAJMJT | Female | II | FHWT |
| TARGET-50-PAJMKI | Male | III | DAWT |
| TARGET-50-PALJIP | Female | II | FHWT |
| TARGET-50-PAJMLI | Male | I | DAWT |
| TARGET-50-CAAAAL | Female | III | FHWT |
| TARGET-50-PAKRVH | Male | II | FHWT |
| TARGET-50-PALDTE | Male | II | FHWT |
| TARGET-50-PAJMVU | Female | IV | DAWT |
| TARGET-50-PAJLWT | Male | II | FHWT |
| TARGET-50-CAAAAB | Female | III | DAWT |
| TARGET-50-PAJNRH | Female | I | DAWT |
| TARGET-50-CAAAAJ | Female | IV | FHWT |
| TARGET-50-PAJNRL | Male | IV | FHWT |
| TARGET-50-CAAAAQ | Female | IV/V | FHWT |
| TARGET-50-PAKGMU | Female | II | DAWT |
| TARGET-50-PADZUB | Female | IV | FHWT |
| TARGET-50-PAKNAL | Female | III | DAWT |
| TARGET-50-PAJPGY | Female | II | FHWT |
| TARGET-50-PAJLIP | Female | III | DAWT |
| TARGET-50-PAKKSE | Female | II | FHWT |
| TARGET-50-PAJNBN | Male | IV | FHWT |
| TARGET-50-PAJMFU | Female | II | DAWT |
| TARGET-50-PAKPDF | Female | III | DAWT |
| TARGET-50-PAKWPM | Male | II | FHWT |
| TARGET-50-CAAAAC | Female | III | FHWT |
| TARGET-50-PAJLKR | Female | IV | FHWT |
| TARGET-50-PAKYLT | Female | III | DAWT |
| TARGET-50-PALFME | Male | II | FHWT |
| TARGET-50-CAAAAH | Female | III | DAWT |
| TARGET-50-PAJNTJ | Male | II | FHWT |
| TARGET-50-PAKFME | Female | III | FHWT |
| TARGET-50-PAJPEW | Male | II | FHWT |
| TARGET-50-PALDWP | Male | III | FHWT |
| TARGET-50-PAJLUJ | Female | IV | FHWT |
| TARGET-50-PALKCW | Male | II | FHWT |
| TARGET-50-PAJPDC | Female | II | FHWT |
| TARGET-50-PAJNVE | Female | III | DAWT |
| TARGET-50-PAJNVX | Male | I | DAWT |
| TARGET-50-PAKJGM | Male | III | FHWT |
| TARGET-50-PAKXXF | Male | III | DAWT |
| TARGET-50-PAJNUS | Male | IV | FHWT |
| TARGET-50-PAKFYV | Female | IIIB | DAWT |
| TARGET-50-PAJLPX | Male | IIIB/V | DAWT |
| TARGET-50-PAJNCC | Male | II | FHWT |
| TARGET-50-PAJNZI | Female | II | DAWT |
| TARGET-50-PAKNRX | Male | II | DAWT |
| TARGET-50-PALEZT | Female | II | DAWT |
| TARGET-50-PAKUIT | Female | IV | DAWT |
| TARGET-50-PAKGZX | Male | IIIB | FHWT |
| TARGET-50-PAKXWB | Female | III | FHWT |
| TARGET-50-PADXAY | Male | II | DAWT |
| TARGET-50-PAKNXS | Male | III | DAWT |
| TARGET-50-PAJNEC | Female | III | FHWT |
| TARGET-50-PAJPAR | Female | III | DAWT |
| TARGET-50-PAJPCM | Female | II | DAWT |
| TARGET-50-PAJMRL | Male | II | DAWT |
| TARGET-50-PALFRD | Female | III | FHWT |
| TARGET-50-PAJNGH | Male | I | FHWT |
| TARGET-50-PAJNZS | Female | III | DAWT |
| TARGET-50-PAKNTW | Male | III | FHWT |
| TARGET-50-PAKKNS | Male | I | DAWT |
| TARGET-50-PAKMSV | Male | III | FHWT |
