# Supplementary material for: Using Elevated Cholesterol Synthesis as a Prognostic Marker in Wilms' Tumor: A Bioinformatic Analysis
Source: Biomed Res Int. 2021 Jan 28;2021:8826286. doi: 10.1155/2021/8826286 (PMC7886595; doi:10.1155/2021/8826286)
Supplement: Supplementary Materials — 1 The gene expression matrix was presented in the supplementary material. 2 All the raw code used in this study were listed as supplementary methods. 3 The baseline characteristics of the patients were summarized in the supplementary. Table 1 The raw data of gender, subtypes, and stage for 130 patients with Wilms tumor was summarized as supplementary Table 2. [file 8826286.f1.zip › supplementary methods.docx]

**Raw code of this analysis.**

1.single sample GSEA

setwd(" ")

inputFile="symbol.txt"

gmtFile="XX.gmt"

library(GSVA)

library(limma)

library(GSEABase)

rt=read.table(inputFile,sep="\t",header=T,check.names=F)

rt=as.matrix(rt)

rownames(rt)=rt[,1]

exp=rt[,2:ncol(rt)]

dimnames=list(rownames(exp),colnames(exp))

mat=matrix(as.numeric(as.matrix(exp)),nrow=nrow(exp),dimnames=dimnames)

mat=avereps(mat)

mat=mat[rowMeans(mat)>0,]

geneSet=getGmt(gmtFile,

geneIdType=SymbolIdentifier())

ssgseaScore=gsva(mat, geneSet, method='ssgsea', kcdf='Gaussian', abs.ranking=TRUE)

normalize=function(x){

return((x-min(x))/(max(x)-min(x)))}

ssgseaOut=normalize(ssgseaScore)

ssgseaOut=rbind(id=colnames(ssgseaOut),ssgseaOut)

write.table(ssgseaOut,file="ssgseaOut.txt",sep="\t",quote=F,col.names=F)

2.COX regression

setwd(" ")

library(survival)

library(forestplot)

options(forestplot_new_page = FALSE)

clrs <- fpColors(box="green",line="darkblue", summary="royalblue")

rt=read.table("expTime.txt",header=T,sep="\t",check.names=F,row.names=1)

outTab=data.frame()

for(i in colnames(rt[,3:ncol(rt)])){

cox <- coxph(Surv(futime, fustat) ~ rt[,i], data = rt)

coxSummary = summary(cox)

coxP=coxSummary$coefficients[,"Pr(>|z|)"]

outTab=rbind(outTab,

cbind(id=i,

HR=coxSummary$conf.int[,"exp(coef)"],

HR.95L=coxSummary$conf.int[,"lower .95"],

HR.95H=coxSummary$conf.int[,"upper .95"],

pvalue=coxSummary$coefficients[,"Pr(>|z|)"])

)

}

write.table(outTab,file="uniCox.xls",sep="\t",row.names=F,quote=F)

rt=read.table("uniCox.xls",header=T,sep="\t",row.names=1,check.names=F)

data=as.matrix(rt)

HR=data[,1:3]

hr=sprintf("%.3f",HR[,"HR"])

hrLow=sprintf("%.3f",HR[,"HR.95L"])

hrHigh=sprintf("%.3f",HR[,"HR.95H"])

3.Survival analysis

library(survivalROC)

setwd(" ")

rt=read.table("lassoRisk.txt",header=T,sep="\t",check.names=F,row.names=1)

pdf(file="ROCw.pdf",width=6,height=6)

par(oma=c(0.5,1,0,1),font.lab=1.5,font.axis=1.5)

roc=survivalROC(Stime=rt$futime, status=rt$fustat, marker = rt$riskScore,

predict.time =5, method="KM")

plot(roc$FP, roc$TP, type="l", xlim=c(0,1), ylim=c(0,1),col='red',

xlab="False positive rate", ylab="True positive rate",

main=paste("ROC curve (", "AUC = ",round(roc$AUC,3),")"),

lwd = 2, cex.main=1.3, cex.lab=1.2, cex.axis=1.2, font=1.2)

abline(0,1)

dev.off()

4. ROC

library(survivalROC)

setwd(" ")

rt=read.table("lassoRisk.txt",header=T,sep="\t",check.names=F,row.names=1)

pdf(file="ROC.pdf",width=6,height=6)

par(oma=c(0.5,1,0,1),font.lab=1.5,font.axis=1.5)

roc=survivalROC(Stime=rt$futime, status=rt$fustat, marker = rt$riskScore,

predict.time =1, method="KM")

plot(roc$FP, roc$TP, type="l", xlim=c(0,1), ylim=c(0,1),col='red',

xlab="False positive rate", ylab="True positive rate",

main=paste("ROC curve (", "AUC = ",sprintf("%.3f",roc$AUC),")"),

lwd = 2, cex.main=1.3, cex.lab=1.2, cex.axis=1.2, font=1.2)

abline(0,1)

dev.off()

4.lasso

rm(list=ls())

library("glmnet")

library("survival")

setwd(" ")

rt=read.table("expTime.txt",header=T,sep="\t",row.names=1)

rt=rt[rt$futime!=0,]

rt$futime=rt$futime/365

gene=read.table("gene.txt",header=F)

rt=rt[,c("futime","fustat",as.vector(gene[,1]))]

x=as.matrix(rt[,c(3:ncol(rt))])

y=data.matrix(Surv(rt$futime,rt$fustat))

fit <- glmnet(x, y, family = "cox", maxit = 1000)

pdf("lambda.pdf")

plot(fit, xvar = "lambda", label = TRUE)

dev.off()

cvfit <- cv.glmnet(x, y, family="cox", maxit = 1000)

pdf("cvfit.pdf")

plot(cvfit)

abline(v=log(c(cvfit$lambda.min,cvfit$lambda.1se)),lty="dashed")

dev.off()

coef <- coef(fit, s = cvfit$lambda.min)

index <- which(coef != 0)

actCoef <- coef[index]

lassoGene=row.names(coef)[index]

geneCoef=cbind(Gene=lassoGene,Coef=actCoef)

write.table(geneCoef,file="geneCoef.txt",sep="\t",quote=F,row.names=F)

riskScore=predict(cvfit, newx = x, s = "lambda.min",type="response")

outCol=c("futime","fustat",lassoGene)

risk=as.vector(ifelse(riskScore>median(riskScore),"high","low"))

outTab=cbind(rt[,outCol],riskScore=as.vector(riskScore),risk)

write.table(cbind(id=rownames(outTab),outTab),

file="lassoRisk.txt",

sep="\t",

quote=F,

row.names=F)
