# Supplementary figures and images for: Using Elevated Cholesterol Synthesis as a Prognostic Marker in Wilms' Tumor: A Bioinformatic Analysis
Source: Biomed Res Int. 2021 Jan 28;2021:8826286. doi: 10.1155/2021/8826286 (PMC7886595; doi:10.1155/2021/8826286)

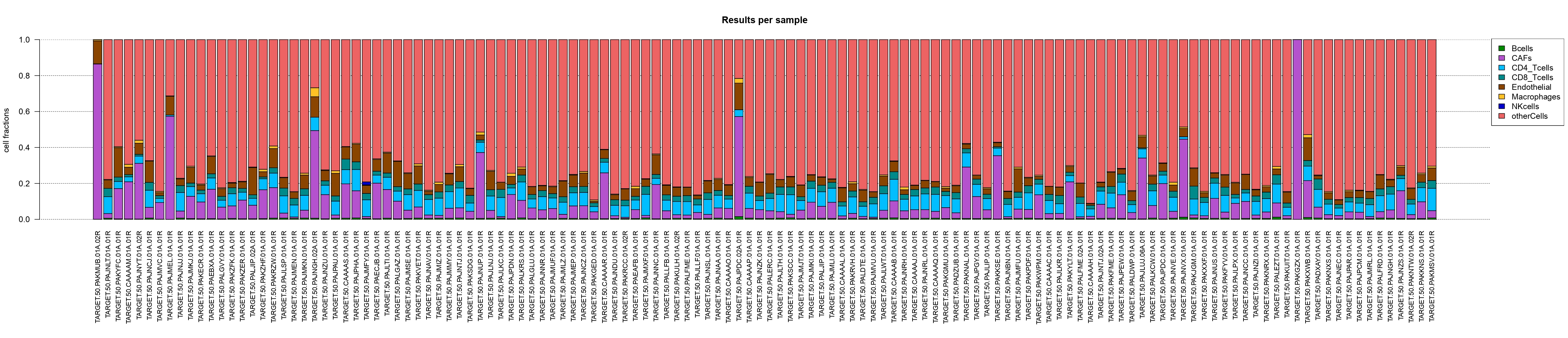

Supplement: Supplementary Materials — 1 The gene expression matrix was presented in the supplementary material. 2 All the raw code used in this study were listed as supplementary methods. 3 The baseline characteristics of the patients were summarized in the supplementary. Table 1 The raw data of gender, subtypes, and stage for 130 patients with Wilms tumor was summarized as supplementary Table 2. [file 8826286.f1.zip › Supplementary Figure 1_page-0001.jpg]

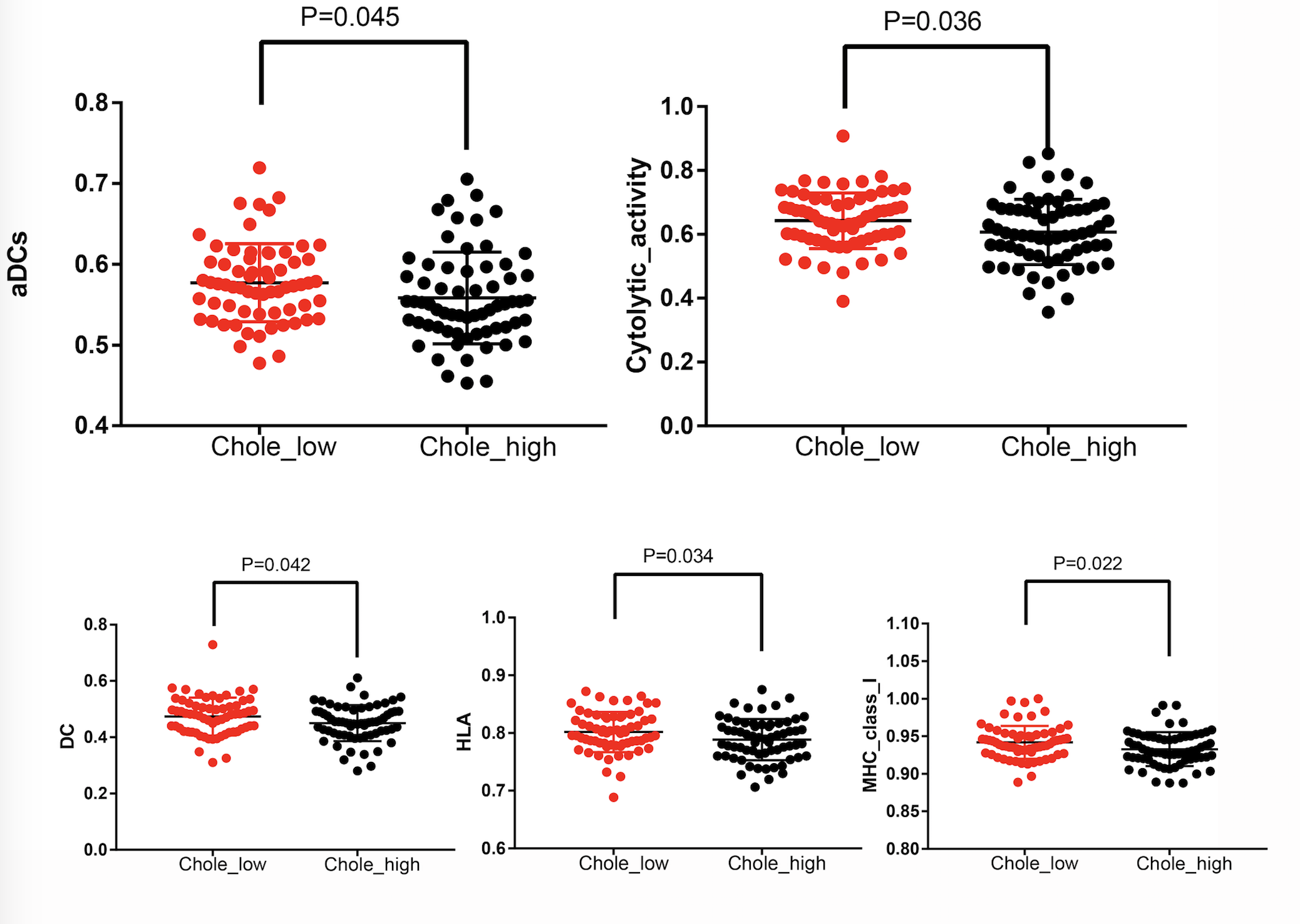

Supplement: Supplementary Materials — 1 The gene expression matrix was presented in the supplementary material. 2 All the raw code used in this study were listed as supplementary methods. 3 The baseline characteristics of the patients were summarized in the supplementary. Table 1 The raw data of gender, subtypes, and stage for 130 patients with Wilms tumor was summarized as supplementary Table 2. [file 8826286.f1.zip › supplementary figure 2 1114.png]
